# Decellularized Rat Lung Extracellular Matrix as an In Vitro Platform for Canine Yolk Sac–Derived Endothelial Precursor Cells for Pulmonary Endothelium Reconstruction Studies

**DOI:** 10.3390/bioengineering13050484

**Published:** 2026-04-22

**Authors:** Leandro Norberto da Silva-Júnior, Maria Angelica Miglino, Bianca de Oliveira Horvath-Pereira, João Victor Barbosa Tenório Fireman, Giovanna Macedo da Siqueira, Maria Laura dos Reis Ferre Pereira, Letícia dos Santos Bezerra, Luís Vicente Franco de Oliveira, Samuel de Sousa Morais, Márcia Zilioli Bellini, Carlos Henrique Bertoni Reis, Rogerio Leone Buchaim, Daniela Vieira Buchaim

**Affiliations:** 1 Graduate Program in Anatomy of Domestic and Wild Animals, Faculty of Veterinary Medicine and Animal Science, University of São Paulo (FMVZ/USP), São Paulo 05508-270, Brazil; silvajunior@alumni.usp.br (L.N.d.S.-J.); bianca.pereira@docente.unievangelica.edu.br (B.d.O.H.-P.); giovannamaccedo12@gmail.com (G.M.d.S.); laura.reis@unesp.br (M.L.d.R.F.P.); lehsantosbr@outlook.com (L.d.S.B.); rogerio@fob.usp.br (R.L.B.); 2 Postgraduate Program in Structural and Functional Interactions in Rehabilitation, Postgraduate Department, University of Marilia (UNIMAR), Marilia 17525-902, Brazil; mariamiglino@unimar.br (M.A.M.); dr.carloshenriquereis@usp.br (C.H.B.R.); 3 Postgraduate Program in Animal Health, Production and Environment, University of Marilia (UNIMAR), Marilia 17525-902, Brazil; 4 Laboratory of Pulmonary and Exercise Immunology (LABPEI), Evangelical University of Goiás (UniEvangélica), Anápolis 75083-515, Brazil; oliveira.lvf@gmail.com; 5 Medical School, University Center of Adamantina (FAI), Adamantina 17800-000, Brazil; 83223@fai.com.br (S.d.S.M.); mzbellini@fai.com.br (M.Z.B.); 6 Postgraduate Program in Sciences, Biomaterials Area, São Paulo State University (FOA/UNESP), Araçatuba 16015-050, Brazil; 7 Beneficent Hospital (HBU), University of Marilia (UNIMAR), Marilia 17525-160, Brazil; 8 Department of Biological Sciences, Bauru School of Dentistry (FOB/USP), University of Sao Paulo, Bauru 17012-901, Brazil; 9 Postgraduate Department, Dentistry School, Faculty of the Midwest Paulista (FACOP), Piratininga 17499-010, Brazil; 10 Postgraduate Program in Clinical Research, Center for Studies of Venoms and Venomous Animals (CEVAP), Faculty of Medicine, São Paulo State University (UNESP), Botucatu 18618-687, Brazil

**Keywords:** lung, reconstruction, recellularization, endothelium

## Abstract

Pulmonary bioengineering holds significant promise for the development of functional lungs suitable for transplantation in patients with terminal lung diseases; however, it encounters considerable challenges. The inherent structural complexity, diverse cellular composition, and the intricate process of re-endothelialization the pulmonary vasculature complicate efforts to reconstruct viable lungs for transplantation. This study aimed to establish an innovative re-endothelialization technique utilizing decellularized scaffolds, integrating canine yolk sac-derived endothelial precursor cells with mechanical respiratory stimuli within a bioreactor framework. Wistar rat lungs were subjected to a decellularization protocol employing SDS + Triton X-100 0.5% and subsequently assessed for cytocompatibility with murine fibroblasts (3T3) and yolk sac (YS) cells in fragments. Following this, the recellularization of the whole-lung scaffold was evaluated under constant mechanical respiratory stimulation with YS cells. Each stage of the process was rigorously analyzed using histological staining, DAPI, scanning electron microscopy (SEM), and genomic DNA quantification. The findings reveal that the implemented alternating decellularization protocol resulted in a structured scaffold conducive to the culture of various cell types in fragments. When subjected to the complete scaffold recellularization model, the results indicated that YS cells are advantageous for the re-endothelialization process. Moreover, when employed in conjunction with the bioreactor model incorporating respiratory stimulation, these cells demonstrated enhanced cellular diffusion capacity and facilitated more homogeneous recellularization of the entire organ. These results signify a notable advancement in the reconstruction of new tissues for pulmonary transplantation.

## 1. Introduction

Chronic obstructive pulmonary disease (COPD) is the third leading cause of death worldwide, accounting for 6% of all deaths [1]. Data demonstrate that respiratory diseases are among the leading causes of death globally, and the number of cases is increasing annually [2,3]. Chronic respiratory diseases can be caused by smoking, exposure to allergens, diseases such as schistosomiasis and sickle cell disease, and high altitude, which may facilitate the emergence of these diseases [4,5,6]. In the advanced stages, pulmonary diseases require lung transplantation as the only indicated treatment because the lung tissue cannot regenerate after substantial injury [7,8]. However, this treatment has a series of complications, such as low organ ischemia time, high rate of post-surgical infections, limited post-transplant survival time, and low demand/supply of viable organs, making this treatment limited [9,10,11].

To meet the demand for transplant organs, pulmonary tissue engineering offers alternatives for the development of biological substitutes through whole decellularized organs as biomimetic alternatives of biological structures for regenerative medicine [12,13]. However, producing a whole lung is challenging because of its complex three-dimensional (3D) structure, cellular diversity, and mechanical relationships during the respiratory process [14,15,16]. Thus, an option for producing a bioartificial lung with well-preserved extracellular matrix is to remove cellular content without disrupting the three-dimensional architecture for subsequent recellularization of these scaffolds, promoting differentiation into lung cells [17,18]. Thus, combining decellularized matrix scaffolds with cells creates bioartificial lungs [19,20].

To obtain organs with transplant potential, various physicochemical protocols are being tested, and the efficiency of such protocols is being evaluated by characterizing the decellularization and preservation of the extracellular matrix [21,22]. The decellularization process consists of the interaction of chemical methods, such as detergent action, physical methods by varying temperature and pressure, and enzymatic methods [23,24]. Decellularization involves the removal of cellular components and genomic material, providing three-dimensional structures that are suitable for tissue reconstitution [25,26,27,28]. Removal of cells from scaffolds reduces the inflammatory reaction and immunological rejection while preserving the natural composition of basic structural proteins and glycosaminoglycans [27,29].

In this context, previous studies have focused on elucidating the molecular mechanisms of lung tissue and their influence on the reconstruction of the pulmonary microenvironment [30,31,32]. Several lung decellularization protocols have been developed to create a structured scaffold that maintains the vascular and airway arrangement [2,33,34]. In most studies, whole lung decellularization was achieved via the perfusion of detergents through the vascular system [35,36,37]. Although research has presented favorable results for pulmonary epithelium reconstitution, the endothelium still presents challenges for functional reconstitution, in addition to reestablishing the gas exchange capacity of the blood-air barrier [38,39,40]. Despite various decellularization methods and cell types for the recellularization of lung scaffolds, no consensus has been established among researchers on the most effective decellularization method and cell type for building bioartificial lungs.

Several characteristics are necessary for a pulmonary scaffold to be used in regenerative medicine. An important aspect to be observed is the physiological function of gas exchange, which must remain intact after decellularization with scaffolds maintaining properties such as strength, elasticity, cellular remodeling, geometry, cell adhesion, and proliferation, as well as nutrient transfer [39,41,42,43,44]. Regarding the bioartificial lungs, the minimum functional unit for scaffold utilization is the blood-air barrier, which is highly specialized between the alveoli and capillaries [38,45,46]. Despite the use of advanced production technologies, such as 3D printing, the complex lung architecture has not yet been successfully reproduced [47,48,49,50,51]. Therefore, acellular lung scaffolds are the most promising approach.

When applying bioengineering to lung tissue, whole scaffolds transformed into hydrogels are already used in recellularization research and drug testing because of the interaction between cells and the extracellular matrix, mimicking the cellular microenvironment [52,53,54,55,56]. This biomaterial is mainly composed of collagens, elastic fibers, glycoproteins (such as fibronectin and laminin), and glycosaminoglycans, among other natural components of the target tissue [17,57].

To promote the construction of complex artificial organs, various cell types are required [39]. Optimizing recellularization and cell maturation protocols is a challenge in acellular lung tissue bioengineering [27,58]. An appropriate cell population capable of performing specific functions is required for the proper repopulation of scaffolds [59,60]. In the recellularization of these scaffolds, cell perfusion protocols and continuous recirculation of the culture medium in bioreactors are used, as well as the use of mechanical stimuli to assist in nutrient distribution and cell diffusion within the scaffold [13,26,37,61,62].

Different cell types have already been employed in attempts at endothelial reconstitution, such as pulmonary endothelial cells [63], Mesenchymal Stem Cells (MSCs) [64,65], Embryonic Stem Cells (ESCs) [66], and Induced Pluripotent Stem Cells (iPSCs) [63,67]. However, there are difficulties related to cell differentiation to obtain pure lineages and cell migration within scaffolds [59]. Another difficulty is directing cell types to different loci within the scaffolds [62]. Thus, the development of techniques to be added to the development of cell differentiation processes during scaffold recellularization may be a solution to make cell reconstitution efficient and optimized. As well as employed in integrated co-culture systems with already differentiated or transfected cells for the secretion of specific molecules to induce differentiation [66,68].

Therefore, this study aimed to evaluate the potential of decellularized rat lung-derived pulmonary scaffolds’ extracellular matrix (ECM) as an in vitro platform for the cultivation and differentiation of endothelial precursor cells derived from the canine yolk sac, with the objective of investigating their capacity to reconstruct pulmonary endothelium. The study seeks to validate the functionality of the decellularized rat lung ECM as a scaffold, focusing on the regeneration of pulmonary endothelial tissues, with an emphasis on the combined effect of the ventilator and bioreactor in enhancing cellular responses, thereby improving the scaffold’s ability to reconstruct pulmonary endothelium.

## 2. Materials and Methods

### 2.1. Lung Samples Acquisition

All experimental procedures involving animals were conducted in accordance with institutional and national guidelines for the care and use of laboratory animals and were approved by the Ethics Committee on Animal Use (CEUA) of the Faculty of Veterinary Medicine and Animal Science, University of São Paulo (CEUAx protocol no. 1824240621). The animals were obtained from the Central Animal Facility of the Evangelical University of Goiás, Anápolis Campus, Brazil, where they were housed under controlled environmental conditions and managed according to established animal welfare protocols. Animal identification and marking procedures were performed by the animal facility staff following institutional guidelines designed to minimize stress and ensure animal well-being. Since the animals were used exclusively for tissue harvesting and were euthanized immediately after organ collection, no longitudinal monitoring or behavioral assessment procedures were required in the present study.

**Figure 1:**
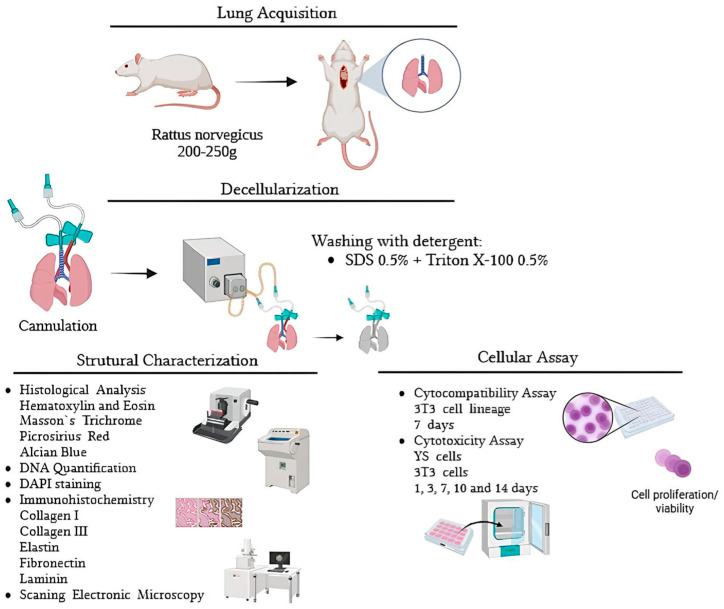
Schematic representation illustrating the methodology employed in this study. Designed using BioRender.com (2 October 2024).

The lungs (*n* = 5 per group) were harvested from adult Wistar rats (*Rattus norvegicus*) weighing between 200 and 250 g. The lung harvesting surgery involved sternotomy, followed by dissection of the trachea up to the larynx, where it was completely severed to enable cannulation using a sectioned scalpel (the outer diameter of the scalpel cannula corresponds to the inner diameter of the adult *Rattus norvegicus* trachea). The heart–lung block was removed carefully. To cannulate the pulmonary artery, a transverse section of the heart was made, and a 24-G cannula was curved and inserted via the right ventricle. After this process, the lungs were immediately frozen at −80 °C until use. This research was conducted in accordance with the institutional regulations of the Ethics Committee of the Faculty of Veterinary Medicine and Animal Science, University of São Paulo (CEUAx protocol no. 1824240621). The methodology is summarized in Figure 1.

### 2.2. Whole-Lungs Decellularization

The lungs were cannulated through the pulmonary artery and trachea and perfused with the assistance of the ORCA Bioreactor™ (Harvard Apparatus, Holliston, MA, USA) perfusion bomb. The decellularization process occurred in three stages: (1) Initial washing—1 h of deionized water (dH_2_O) and 1 h of 1X phosphate-buffered saline (PBS) at a tracheal rate of 2 mL/min and arterial rate of 4 mL/min, with tracheal perfusion pressure of 10 cmH_2_O and arterial perfusion pressure of 20 cmH_2_O. (2) Decellularization—0.5% sodium dodecyl sulfate (SDS) + 0.5% Triton X-100 for 5 h following the same perfusion rate as in stage 1. (3) Final washing—deionized water for 6 h and 1% PBS for 6 h to remove detergents. The pulmonary scaffolds were stored in 1X PBS at 4 °C for further analyses.

Perfusion was conducted through both the pulmonary artery and the tracheal route, with half of the perfusion time performed through each pathway. This strategy was adopted to improve the penetration of the detergent solution into the lung parenchyma and to ensure effective exposure of both vascular and airway structures to the decellularization process. For clarity and reproducibility, the complete decellularization procedure, including solutions, concentrations, perfusion routes, durations, and flow/pressure parameters, is summarized in Table 1.

### 2.3. Histological Analysis

Histological evaluation was employed to confirm the efficacy of the decellularization protocol and to assess the morphological integrity of ECM components. Lung fragments, both native and decellularized, were fixed in 4% buffered paraformaldehyde (PFA 4%) for 48 h. Subsequently, they underwent dehydration in a series of increasing alcohol concentrations (70%, 80%, 90%, and 100%), cleared in xylene, and embedded in paraffin. Sections with a thickness of 5 µm (No. RM2265; Leica, Wetzlar, Germany) were used for various staining’s such as Hematoxylin & Eosin (H&E) to evaluate the presence of nuclei and the overall morphological condition of the extracellular matrix (ECM); Masson’s Trichrome to detect total collagen content; Alcian Blue (pH = 2.5) to evaluate the overall glycosaminoglycan (GAG) content; and Picrosirius Red to distinguish different stages of collagen maturation. Slides were photographed and subjected to analysis using a light microscope (Nikon ECLIPSE 80I, Nishi-Õi, Japan, CADI FMVZ-USP).

### 2.4. 4,6-Diamidino-2-fenilindole (DAPI) Staining

DAPI fluorescent staining was utilized to confirm the presence of nuclei or DNA fragments in the scaffolds post-decellularization process. Specimen fragments (both native and decellularized) were cryopreserved in Tissue Plus O.C.T. compound optimal cutting temperature (Fisher Health Care, Houston, TX, USA) and sectioned into microsections using a cryostat (CM1860 model, Leica Biosystems, Baden-Wurttemberg, Germany). The slides were stained with DAPI solution (1:10,000 dilution) at room temperature in the absence of light for 10 min, followed by washing with 1X PBS for subsequent analysis using fluorescent microscopy (Nikon ECLIPSE 80I, CADI FMVZ-USP, Tokyo, Japan).

### 2.5. Genomic DNA Quantification

To quantify genomic DNA, the QIAamp^®^ DNA Mini kit (Qiagen, Hilden, Germany) was used to extract genetic material from native, decellularized and recellularized samples following the manufacturer’s instructions. The fragments were subjected to overnight digestion at 56 °C using Proteinase K stabilized in the kit’s lysis buffer. Subsequently, the fragments were purified and subjected to spectrophotometric analysis at 260 nm using the Nanodrop (Thermo Scientific, Waltham, MA, USA).

### 2.6. Scanning Electronic Microscopy (SEM)

The native and decellularized fragments were fixed in Karnovsky solution (2.5% glutaraldehyde and 4% paraformaldehyde in a buffered 0.1 M sodium cacodylate) for 48 h and dehydrated in increasing concentrations of alcohol for 5 min at each concentration. Then, the fragments were dried using a supercritical point device (LEICA EM CPD 300^®^, Wetzlar, Germany) and subsequently coated with gold spray (EMITECH K550^®^, Quorum Technologies, East Sussex, UK). Finally, the samples were photographed using a scanning electron microscope (LEO 435 VP^®^, Oberkochen, Germany).

### 2.7. Immunohistochemistry Analysis

First, sections of native and decellularized tissues were rehydrated in citrate buffer in a pressure cooker for 5 min for antigen retrieval. Then, endogenous peroxidase activity was blocked with 3% hydrogen peroxide in distilled water for 30 min in the dark. Subsequently, a 2% bovine serum albumin (BSA) solution in PBS was used to block nonspecific protein interactions. The primary antibodies chosen were anti-collagen I (#PA5-29569, 1:250, Invitrogen, Carlsbad, CA, USA), anti-collagen III (#PA1-28870, 1:250; Invitrogen), anti-elastin (#Ab9519, 1:100, Abcam), anti-fibronectin (#Ab2413, 1:100, Abcam, Cambridge, UK), anti-laminin subunit α2 (#PA1-16730, 1:200; Invitrogen), and the secondary antibodies were IgG anti-mouse/anti-rabbit (#K800; Dako, Carpinteria, CA, USA). Overnight incubation occurred in a wet chamber at 4 °C. The reaction was detected by Dako Advance HRP (#K6068; Dako) and revealed with DAB (#k3468; Dako), according to the manufacturer’s instructions. Subsequently, slides were photographed and subjected to analysis using a light microscope (Nikon ECLIPSE 80I, Nishi-Õi, Japan, CADI FMVZ-USP).

### 2.8. Scaffolds Sterilization

At the end of the decellularization and washing process, whole lung scaffolds were perfused for 5 min with 70% alcohol. For sterilization, both the scaffolds to be fragmented (*n* = 5) and the whole scaffolds (*n* = 10) were placed inside the laminar flow hood. The scaffolds were fragmented and immersed in a progressive and regressive alcohol curve at concentrations of 70%, 80%, 90%, 100%, 90%, 80%, and 70% for 5 min at each concentration. For the whole scaffolds, with the aid of a syringe, alcohol at concentrations of 70%, 80%, 90%, 100%, 90%, 80%, and 70% was perfused through the arterial and tracheal routes for 5 min at each concentration. After the immersion cycles in alcohols, the fragments were washed 5 times with 1X PBS containing 2% antibiotic (Penicillin-Streptomycin 10,000 µg/mL, LGC Biotecnologia, Cotia, Brazil), and the whole lungs were perfused for 10 min through each cannulation route with PBS and antibiotic to remove all alcohol from the scaffolds. After PBS washing, both the fragments and the whole scaffolds were exposed to UV light for 5 min and then stored in a PBS solution with 2% antibiotic for future analysis. As a method of evaluation and confirmation of sterilization, fragments were immersed in DMEM High culture medium (Sigma, St. Louis, MO, USA) supplemented with 10% bovine serum (Invitrogen Co., Ltd., Carlsbad, CA, USA) and placed in a culture incubator at 37.5 °C and 5% CO_2_ for 7 days.

### 2.9. Microbiological Evaluation

For the microbiological evaluation, 100 µL of the cell culture medium (DMEM) obtained from the scaffolds that had been immersed for 96 h to assess sterilization efficacy and from the recellularization process was analyzed. The samples were inoculated into Luria–Bertani (LB) liquid and solid media (agar) following the Brazilian Pharmacopeia guidelines. They were then incubated in an orbital shaker (Ética) at 250 rpm at 37 °C for 9 days in the liquid medium, while solid LB plates were kept at room temperature (22–24 °C) for 15 days. After the incubation period, the culture tubes and plates were examined for turbidity and the emergence of microbial colonies, which would indicate potential contamination.

### 2.10. Cytotoxicity Assay

Cell viability was evaluated over a 14-day culture period using the resazurin assay. Measurements were performed at days 1, 3, 7, 10, and 14, allowing evaluation of the temporal progression of cell metabolic activity and proliferation in contact with the decellularized lung scaffolds. To assess the cell viability of the biomaterials, the resazurin assay was performed. Initially, endothelial precursor cells derived from the canine yolk sac (YS), provided by Professor Maria Angelica from the University of Marília [69], and murine fibroblast cells (3T3) were cultured in DMEM High medium supplemented with 10% fetal bovine serum (FBS, Gibco, Paisley, UK) and antibiotics (Penicillin-Streptomycin 10,000 µg/mL, LGC Biotecnologia, Cotia, Brazil). Cultures were maintained at 37 °C and 5% CO_2_ for 24 h. Then, 3T3 and YS cells were trypsinized, and a cell density of 2 × 10^3^ cells/mL was cultured on the lung scaffolds in a 24-well plate. Subsequently, 1 mL of resazurin solution (0.14 mg/mL in PBS; Thermo Fisher, Waltham, MA, USA) was added to each well. For analysis, 200 μL of the incubation medium was collected on days 1, 3, 7, 10, and 14 of culture and stored in a 96-well culture plate (KASVI-K12-096). The collected samples were stored in the refrigerator until the day of analysis (day 14). On the final day of the experiment (D14), samples were analyzed using a spectrophotometer (uQuant, Bio-Tek Instruments, Inc., Winooski, VT, USA) at a wavelength of 540 nm. The control group underwent the same culture conditions as the cells cultured without the presence of lung scaffolds. The data obtained from these measurements were then used to generate proliferation graphs.

### 2.11. Cytocompatibility Assay

To evaluate scaffold cytocompatibility and early cell adhesion, 3T3 and YS cells were cultured on decellularized lung scaffold fragments for 7 days. After this period, the samples were analyzed using histology, DAPI staining, and scanning electron microscopy (SEM) to assess cellular attachment and distribution on the extracellular matrix surface. The scaffold viability test was adapted from [70]. For this, the sterilized scaffold fragments were placed in a 24-well culture plate without cell adhesion treatment (TC-Platte 24, Well, Suspens., F from SARSTEDT, Numbrecht, Germany), where they remained for 7 days incubated at temperature of 37.5 °C and 5% CO_2_, the organization of the wells followed the following conformation: 11 wells containing complete culture medium (90% Modified MEM Alpha Culture Medium from LGC Biotechnology + 10% fetal bovine serum with addition 2% Penicillin) + scaffold + 3T3 cells in the quantity of 5 × 10^3^; 2 wells with medium + cells; 2 wells with complete medium + scaffold; 2 wells containing complete medium only. At the end of the process, the wells were photographed, and the fragments were fixed according to the analysis to which they were submitted.

### 2.12. Recellularization of the Whole-Lungs Scaffolds

**Figure 2:**
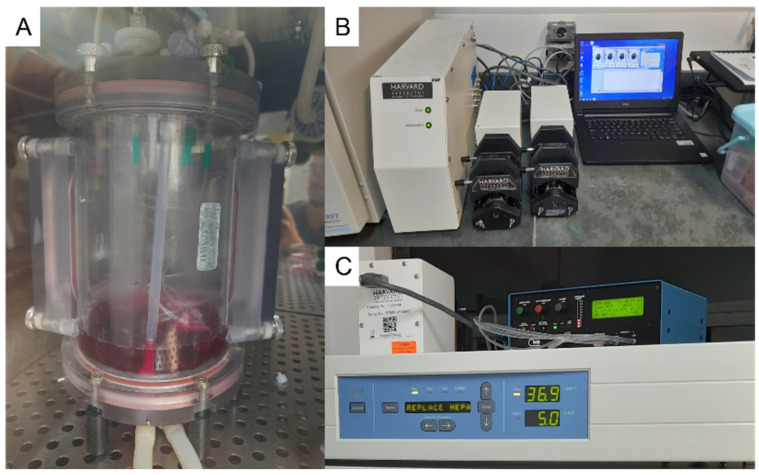
Final reendothelialization system. (**A**). Displays the culture chamber of the Orca bioreactor, containing the lungs undergoing reendothelialization. (**B**). Shows the control system and perfusion pump of the Orca bioreactor, which regulate the flow and conditions for the process. (**C**). Presents the SAR-1000 ventilator and perfusion pump, both essential for providing cyclic stretching and maintaining perfusion during the reendothelialization procedure, ensuring proper gas exchange and mechanical stretching to mimic physiological conditions.

The bioreactor assembly employed a Small Animal Ventilator SAR-1000 (CWE, Inc., Ardmore, PA, USA) to facilitate constant cyclic stretching through the airway. To ensure continuous perfusion of the culture medium, the Perfusion Pump of the Orca Bioreactor from Harvard Apparatus™ was utilized, interfacing with the pulmonary arterial flow. These devices were incorporated into the culture system within a Thermo Scientific™ Forma™ Series 3 Water Jacketed CO_2_ incubator, as illustrated in Figure 2.

Initially, all materials were properly sterilized and assembled within the culture hood. With the scaffold already connected to the system, an initial perfusion of DMEM high-glucose culture medium supplemented with 10% fetal bovine serum and 1% penicillin-streptomycin was conducted. The culture environment was maintained at 37.5 °C and 5% CO_2_. The whole-lung recellularization experiment was conducted over a total period of 9 days, consisting of three sequential phases. First, an initial stabilization phase (24 h) was performed, during which the decellularized scaffolds were perfused and maintained in culture medium without cells to allow equilibration of the system and stabilization of the biomaterial within the bioreactor environment. Second, cell seeding was performed by injecting 1 × 10^6^ canine yolk sac–derived endothelial progenitor cells, obtained from a previously established and characterized cell bank (Fratini et al., 2016 [69]), into the scaffolds. After cell perfusion, the scaffolds were maintained under static conditions in culture medium for 24 h to promote initial cellular attachment to the extracellular matrix. Finally, a dynamic recellularization phase lasting 7 days was initiated using mechanical ventilation and continuous medium perfusion within the bioreactor system. Mechanical breathing was maintained at a frequency of 35 rpm, with culture medium circulation through the arterial route at a flow rate of 1 mL/min, while maintaining a maximum perfusion pressure of 20 cmH_2_O. The experiment lasted for a total of 9 days. As a control, recellularization of three scaffolds with a similar protocol was performed simultaneously, excluding the mechanical breathing system. The recellularized scaffolds were analyzed using the histological techniques described in previous sections.

### 2.13. Statistical Analysis

Statistical analysis was performed using GraphPad Prism software (version 7.00, GraphPad Software, San Diego, CA, USA). Data are presented as mean ± standard deviation (SD). Cell viability data obtained from the resazurin assay were analyzed using two-way analysis of variance (Two-way ANOVA) with repeated measures, considering time (days 1, 3, 7, 10, and 14) and cell type (3T3 and YS cells) as independent factors. When significant differences were detected, Tukey’s multiple comparisons test was applied as a post hoc analysis to determine pairwise differences between groups. Statistical significance was considered at *p* < 0.05.

## 3. Results

### 3.1. Whole-Lung Perfusion Decellularization

**Figure 3:**
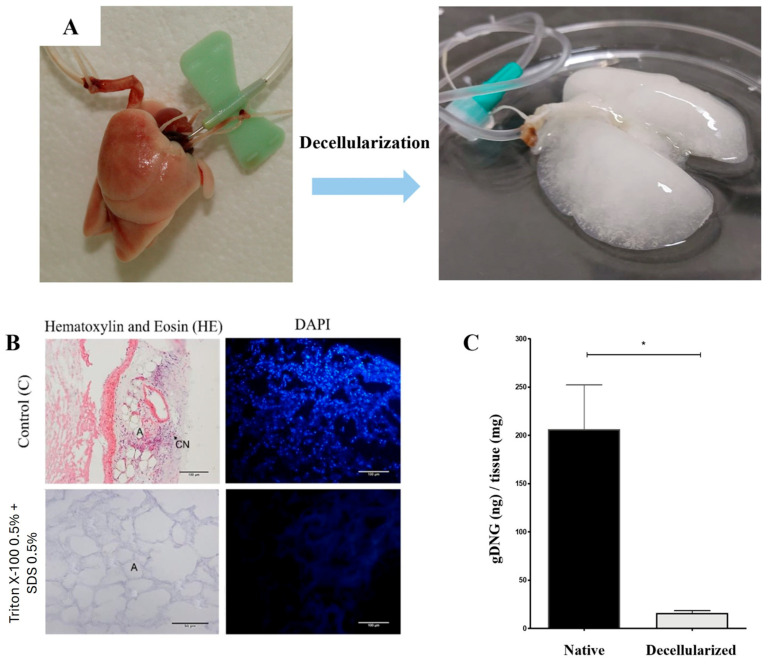
Whole-lung perfusion decellularization efficiency evaluation. (**A**) Whole native lung cannulated via trachea and pulmonary artery, alongside the macroscopic appearance of perfusion-decellularized lungs, demonstrating progressive whitening as a result of cellular removal through continuous perfusion. (**B**) Hematoxylin and Eosin staining and DAPI nuclear fluorescence comparing native and decellularized lungs. (**C**) Quantification of genomic DNA from native and decellularized rat lungs. *n* = 3. * (*p* < 0.05).

Cannulation was performed through two pathways: the pulmonary artery, which was dissected and cannulated using a 24-G needle securely tied and stabilized with nylon thread, and the tracheal via, where the trachea was cannulated with a 24G needle similarly secured with nylon thread. Throughout each stage of the perfusion process (dH_2_O, PBS, detergents, and final wash), the lungs exhibited progressive clearing until the final stage, where they became completely translucent and white, indicative of successful decellularization (Figure 3A). The primary parameter used to assess the decellularization process was the macroscopic appearance of the lungs.

Histological analysis revealed, through H&E and DAPI staining, that compared to native tissue, the alternating perfusion of SDS and Triton X-100 effectively removed cells while preserving the structural integrity of the lung extracellular matrix (ECM) (Figure 3B). These results were further corroborated by DNA quantification, which demonstrated a significant reduction of 98.8% in DNA content compared to native tissue, confirming the efficacy of the decellularization protocol (Figure 3C).

### 3.2. Morphological and Ultrastructural Characterization

**Figure 4:**
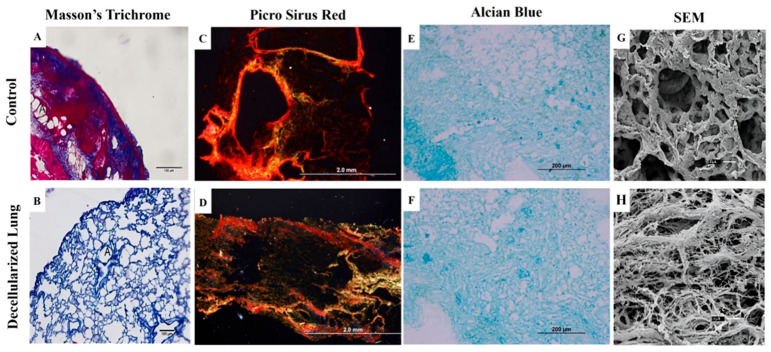
Histological analysis of the ECM from native and decellularized lungs. Hematoxylin and eosin staining indicates the general tissue structure and the presence or absence of cells in native and decellularized samples. (**A**,**B**) Masson’s trichrome staining highlights the total collagen content in blue in both native and decellularized samples. (**C**,**D**) Polarized Picrosirius red staining distinguishes mature collagen (red and yellow) from immature collagen (green). (**E**,**F**) Alcian blue staining reveals GAG content in native and decellularized samples. (**G**,**H**) Representative scanning electron micrographs of native and decellularized lung tissue.

The analyses enabled a comprehensive morphological and three-dimensional characterization of the resulting biomaterials. Masson’s Trichrome staining revealed the preservation of total collagen content in the decellularized samples, with collagen fibers staining blue (Figure 4B), without any apparent disorganization of the collagen fiber bundles compared to native tissue (Figure 4A). Additionally, Masson’s Trichrome staining supported earlier findings of successful cellular removal, as no dark or black-stained nuclei were evident in the decellularized samples, which were present in the native tissue.

In lung tissue, collagen fibers display varied arrangements across different compartments, reflecting distinct levels of maturation throughout the tissue composition. To further investigate these different stages of collagen fiber arrangement, Picrosirius Red staining was employed, staining total collagen (Figure 4C,D). Under polarized light, this staining distinguished mature collagen (reddish and yellowish hues) from immature collagen (greenish hues) based on birefringence. Alcian Blue staining demonstrated that both groups exhibited a preserved pattern of glycosaminoglycan content, especially in vascular regions (Figure 4E,F), consistent with the three-dimensional characteristics observed through scanning electron microscopy (SEM) in both native and decellularized lung samples (Figure 4G,H).

**Figure 5:**
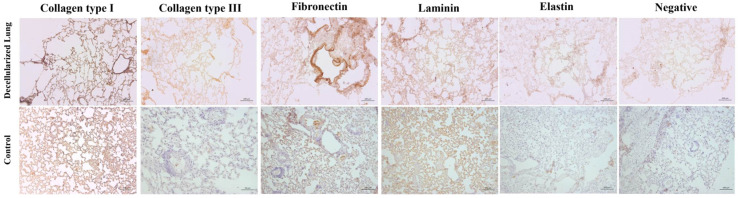
Immunohistochemical analysis of the main ECM components of the native and decellularized lungs. Immunolocalization of type I collagen, type III collagen, fibronectin, elastin and laminin in native and decellularized samples.

Immunohistochemical analyses were conducted to verify the presence of main extracellular matrix (ECM) components following the decellularization procedure (Figure 5). Immunohistochemical analysis revealed that Type I and III collagen were retained in the decellularized tissues, exhibiting strong positive staining patterns comparable to those observed in native samples. Similarly, fibronectin demonstrated significant immunopositivity in large vessels and alveolar septa, indicating that this critical extracellular matrix (ECM) component remained intact following the decellularization process. Laminin maintained its presence within the vascular structures and alveolar compartments, suggesting minimal loss of basement membrane proteins after decellularization. Additionally, elastin, an essential component of elastic fibers, was preserved, with staining patterns in decellularized tissues closely mirroring those of native samples. These findings collectively demonstrate that the primary ECM proteins, including Type I and III collagen, fibronectin, laminin, and elastin, remained intact and were not significantly affected by the decellularization process. This preservation is crucial for maintaining the scaffold’s biological relevance, particularly for potential recellularization and tissue engineering applications.

### 3.3. Microbiological Evaluation

Microbiological evaluation was performed to confirm the sterility of the decellularized scaffolds and the culture conditions used during the recellularization experiments. No turbidity was observed in the liquid cultures and no microbial colonies were detected on LB agar plates during the incubation period. These results indicate that the sterilization protocol applied to the scaffolds was effective and that both the decellularized scaffolds and the culture media used in the recellularization experiments with YS cells remained free of microbial contamination.

### 3.4. Cytotoxicity Evaluation

**Figure 6:**
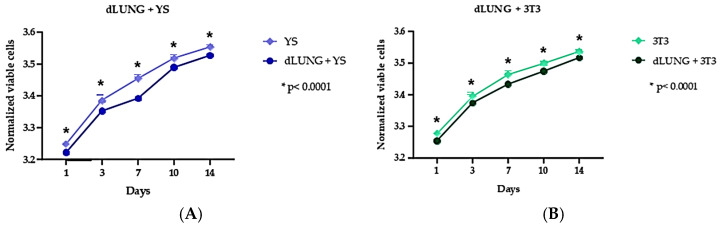
Cell viability assessment of YS and 3T3 cells cultured on decellularized lung after 1, 3, 7, 10 and 14 days. Trend graphs of YS cells (**A**) and 3T3 cells (**B**). * (*p* < 0.0001).

Cell viability was evaluated over a 14-day culture period using the resazurin assay. During this experiment, two cell types, murine fibroblasts (3T3) and canine yolk sac–derived endothelial precursor cells (YS), were cultured on pulmonary scaffolds (Figure 6). Metabolic activity was measured at days 1, 3, 7, 10, and 14 through the reduction in resazurin, a purple compound that is converted by mitochondrial activity into resorufin, a fluorescent pink molecule. This color transformation was utilized as an indicator of cellular viability [71]. A similar growth pattern was observed for both YS (Figure 6A) and 3T3 cells (Figure 6B). During the 14-day experimental period, the cells remained viable in contact with the scaffolds, demonstrating good cytocompatibility. From day 1 to day 14, an increase in cell proliferation was noted for both cell types employed. Mitochondrial metabolism, as evidenced by the oxidation of resazurin, remained consistent and significant, as illustrated in the trend graphs (Figure 6). The results indicated that the generated scaffolds allowed satisfactory adherence and survival of various cell types, suggesting their potential for use as platforms for in vitro cell culture and tissue reconstruction.

### 3.5. Cytocompatibility Evaluation

A scaffold–cell adhesion assay was performed to evaluate the adhesion ability of 3T3 and YS cells to decellularized pulmonary scaffolds, a crucial factor for effective tissue integration and regeneration. Results showed that scaffolds treated with SDS and 0.5% Triton X-100 enabled cell adhesion, indicating their capacity to support cell attachment and subsequent tissue reconstitution. The selection of 3T3 and YS cells aimed to demonstrate the scaffold’s non-cytotoxic properties while evaluating their ability to support non-pulmonary-derived cells. Scaffold fragments seeded with 3T3 or YS cells were cultured for 7 days, after which cellular adhesion and distribution were evaluated through histological analysis, DAPI staining, and scanning electron microscopy.

**Figure 7:**
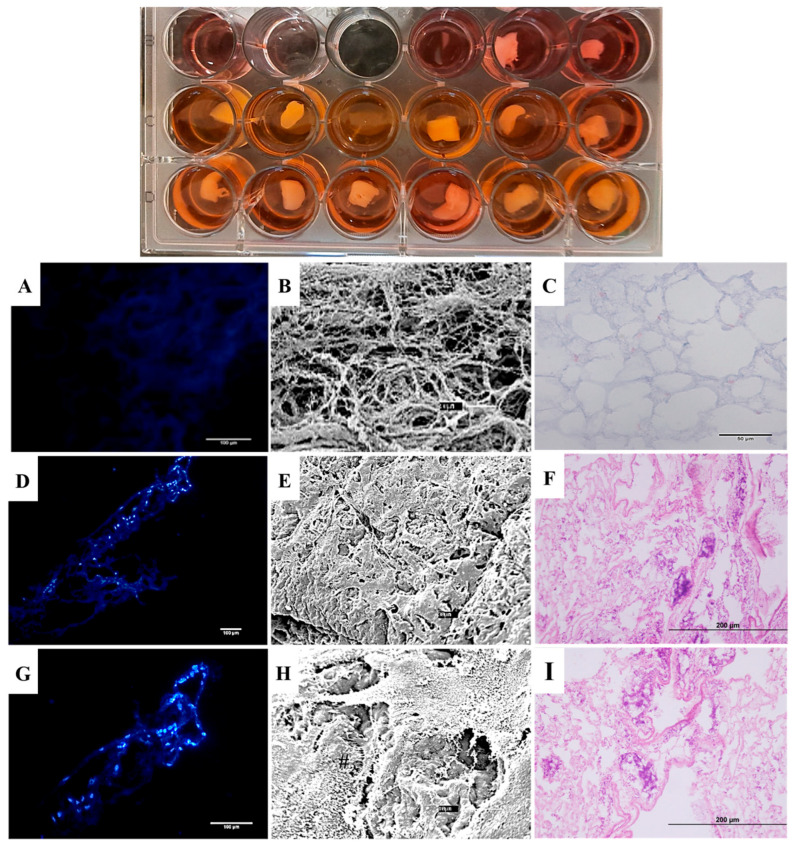
DAPI staining (**A**,**D**,**G**); SEM microscopy (**B**,**E**,**H**); and H&E staining (**C**,**F**,**I**) comparing decellularized scaffolds (**A**–**C**), scaffolds cultured for 7 days with 3T3 cells (**D**–**F**), and scaffolds cultured for 7 days with YS cells (**G**–**I**).

Hematoxylin and Eosin (H&E) (Figure 7C,F,I) and DAPI (Figure 7A,D,G) staining was performed, confirming cell attachment and distribution on the scaffold surface. SEM analysis revealed that the cells adhered to the extracellular matrix (ECM) via membrane projections, facilitating interaction across the scaffold surface (Figure 7B,E,H). These findings suggest that the scaffolds effectively support cell adhesion and proliferation, likely due to the retention of adhesive glycoproteins in the ECM, which enable cellular attachment and interaction.

**Figure 8:**
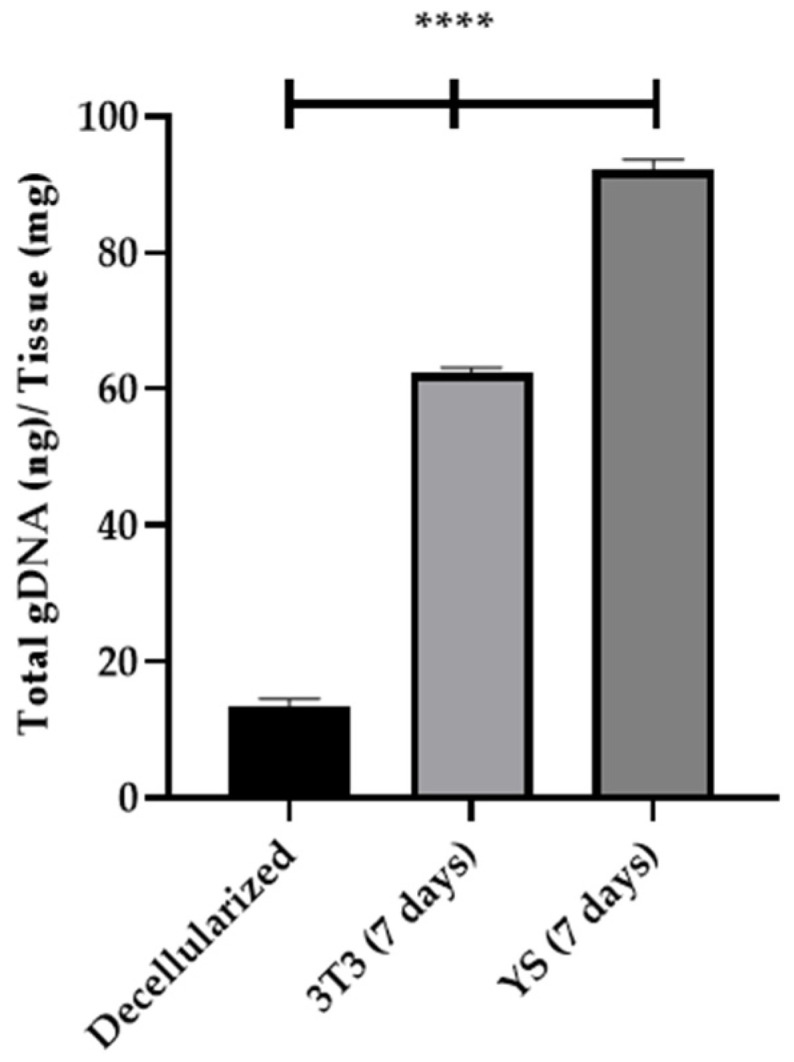
Quantification of Genomic DNA from Recellularized Lung Scaffolds (7 Days) with 3T3 and YS cells (****, *p* < 0.0001).

By quantifying the genomic DNA of recellularized lung scaffolds over a 7-day period (Figure 8), a significant increase in the presence of 3T3 and YS cells during recellularization was demonstrated, compared to the values observed following scaffold decellularization using the SDS + Triton 0.5% protocol (13.44 ng/mg of gDNA). This suggests that the recellularization process effectively promoted cellular proliferation and integration within the scaffold. Specifically, the levels of genomic DNA, which serve as a proxy for the number of cells within the scaffold, showed a progressive and substantial rise, indicating successful colonization and adhesion of both 3T3 (62.38 ng/mg of gDNA) and YS (92.12 ng/mg of gDNA) cells. These results highlight the potential of the recellularized scaffolds to promote and maintain cellular proliferation and viability, suggesting that the applied decellularization protocol not only preserved the structural integrity of the lung matrix but also maintained its bioactive properties, essential for subsequent cell attachment and proliferation. The differences between the recellularized scaffolds and the decellularized controls further confirm the efficacy of the recellularization process in restoring the biological functionality of the matrix.

Whole lung recellularization experiments were conducted for 9 days, including 24 h of system stabilization, 24 h of static cell adhesion, and 7 days of dynamic culture under mechanical ventilation. A scaffold–cell adhesion assay was conducted on whole decellularized lungs using YS cells to assess the effectiveness of two conditions: dynamic breathing in a ventilated bioreactor versus a static supine control. The dynamic breathing of the scaffold, mimicking physiological respiration, significantly enhanced the uniformity of cell distribution and adhesion compared to static systems. The continuous respiratory motion facilitated better nutrient and oxygen delivery, as well as waste removal, optimizing the recellularization process.

**Figure 9:**
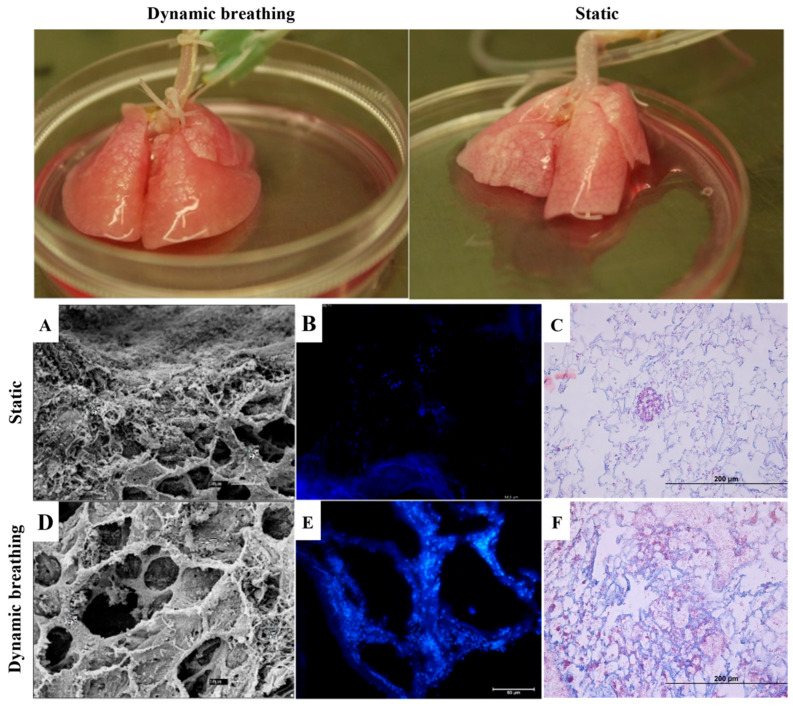
Experimental design for the recellularization of lung structures under static and dynamic respiration. The analysis of recellularization conditions was performed using Scanning Electron Microscopy (SEM) (**A**,**D**), DAPI staining (**B**,**E**), and Masson’s Trichrome staining (**C**,**F**).

Histological analysis through Masson’s trichrome staining (Figure 9C,F) in the ventilated condition showed more widespread and uniform cell integration throughout the scaffold, particularly in regions with higher air exposure. In contrast, the supine static lung exhibited less uniform cellular coverage and reduced scaffold infiltration. DAPI staining (Figure 9B,E) confirmed that the breathing lungs had more evenly distributed nuclei across the scaffold, while the static condition presented with patchy and less consistent cell adhesion.

SEM analysis (Figure 9A,D) revealed that cells within the ventilated lungs formed well-defined membrane projections, establishing consistent interactions with the extracellular matrix (ECM). The dynamic movement induced by the ventilator appeared to enhance cytoskeletal organization, resulting in more extensive surface coverage by the cells. Additionally, the cells in the ventilated scaffolds showed more uniform spatial distribution, aligning along the scaffold architecture and interacting effectively with its fibers. In contrast, the supine controls displayed reduced cellular contact with the ECM, with fewer interactions between cells and the scaffold, indicating lower efficiency in achieving uniform recellularization. These results demonstrate the role of mechanical stimulation in facilitating the integration of cells into decellularized lung scaffolds.

The improved performance of the ventilated lungs can be attributed to the cyclic respiratory movements, which improved oxygen and nutrient distribution, as well as waste removal, supporting better cellular adhesion and integration.

Additionally, the use of endothelial precursor cells from yolk sac (YS) in this experiment was crucial for the re-endothelialization process. These cells, which are precursors to endothelial cells, play a key role in forming the vascular lining, an essential component in pulmonary tissue regeneration. This approach highlights the importance of combining appropriate cellular types with optimized mechanical stimuli to improve the success of lung recellularization strategies for future pulmonary therapies.

Quantification of genomic DNA from whole recellularized lungs, specifically utilizing the static supine and dynamic breathing supine YS models over a 14-day period, revealed a significant increase in the presence of YS cells during the recellularization process. Notably, the dynamic breathing supine YS model demonstrated superior results compared to the static supine YS model.

**Figure 10:**
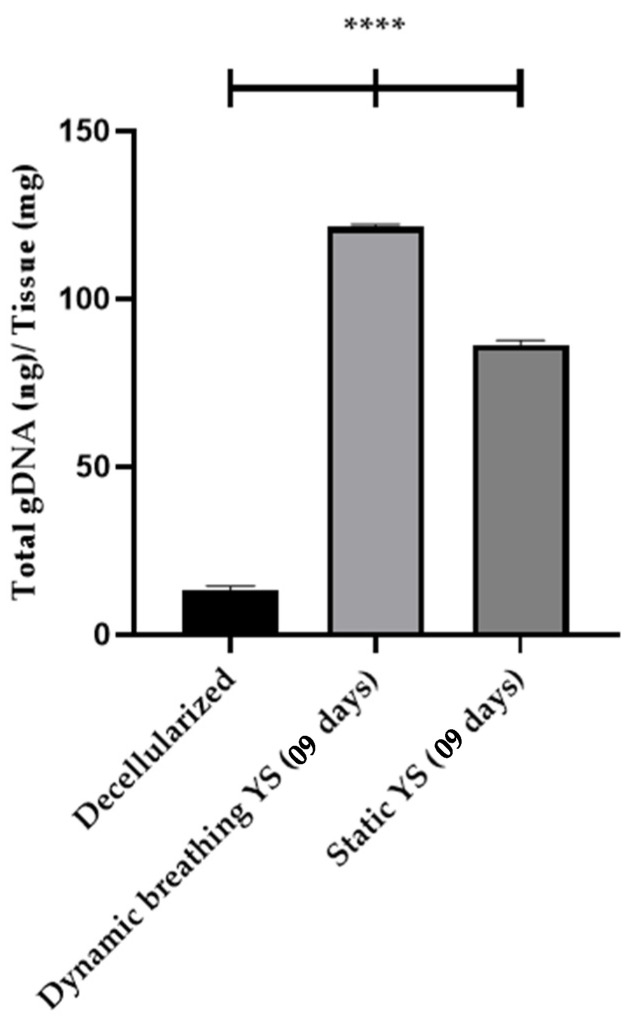
Quantification of Genomic DNA from Recellularized Whole-Lung Scaffolds (09 Days) with YS Cells under Dynamic Breathing and Static Conditions (****, *p* < 0.0001).

The levels of genomic DNA, serving as a quantitative measure of cellular presence, exhibited a marked increase in the dynamic breathing group (121.50 ng/mg of gDNA) when compared to the static supine group (86.33 ng/mg of gDNA), indicating enhanced cellular proliferation and integration within the lung scaffold matrix. These findings underscore the efficacy of dynamic breathing in promoting successful colonization and adhesion of YS cells (Figure 10).

## 4. Discussion

The detergent perfusion technique requires precise cannulation of the selected vessel through catheter insertion, thereby enabling effective diffusion of the solution throughout the organ [72]. The pulmonary circulation is unique both in volume and function, as it is the only organ with two types of circulation: the pulmonary circulation, which is responsible for gas exchange, and the bronchial circulation, which supplies oxygenated blood to the walls of the conducting airways, pulmonary arteries, and veins [73].

Decellularized lung scaffolds have been described as promising platforms for in vitro cell cultivation for the treatment of pulmonary diseases [74,75,76,77]. There are numerous limitations in lung tissue engineering and regenerative medicine due to the lung’s complex structure [78]. Many studies have attempted to produce complex lung structures from biomaterials, but this approach has several limitations. Recently, decellularization has emerged as an alternative for obtaining biomimetic decellularized scaffolds to support differentiated stem cells, representing an important approach for treating pulmonary diseases. Therefore, researchers have been striving to replicate this complex three-dimensional architecture using a decellularized scaffold [30,79,80,81,82].

In recent years, several lung decellularization techniques have been developed with the aim of preserving extracellular matrix (ECM) proteins and achieving three-dimensional architecture. The commonly used approach involves detergent-based decellularization using substances such as SDS, Triton X100, sodium deoxycholate (SDC), and 3-[(3-~holamidopropyl) dimethylammonio]-1-propane-sulfonate (CHAPS) [77,83,84]. However, an ideal method for decellularizing lungs while maintaining their architecture is still unclear. Additionally, there is no optimized protocol in the literature for lung decellularization that preserves the three-dimensional structure and ECM proteins [85,85,86,87]. Therefore, although various decellularization methods exist, no consensus has been established regarding the best method.

In this study, a decellularization protocol was presented to generate a biological acellular scaffold of the lung based on previous studies that used SDS in combination with Triton X-100. Triton X-100 (non-ionic) and SDS (ionic) are two common detergents used in the decellularization process [88,89]. The choice of these detergents was due to Triton X-100’s ability to disrupt lipid–protein and lipid–lipid interactions, but not protein–protein interactions, resulting in the separation of cells and the release of cytoplasmic materials due to cell membrane lysis [90,91]. In decellularization methods, non-ionic detergents are generally preferred due to their milder effects on tissue structure; however, their efficiency in removing cellular components is low [92,93]. SDS, on the other hand, has the ability to denature proteins by disrupting protein–protein interactions while preserving the structure and composition of the extracellular matrix (ECM), which makes it effective in removing cellular residues [94,95]. Due to these characteristics, we opted to combine both detergents at a concentration of 0.5% to obtain an acellular lung scaffold with preserved ECM components in this study.

Thus, we present a new decellularization protocol for generating an acellular biological scaffold from the entire rat lung using perfusion with 0.5% SDS and 0.5% Triton X-100 detergents via the Harvard Apparatus: ORCA. The perfusion of detergents through the vascular system with a controlled flow rate allows the solutions to easily penetrate the organ’s parenchyma, reaching regions with little surface contact that are otherwise difficult to access [44,61,62,72,96]. This technique also preserved macro- and microstructural integrity [96]. Histological and immunohistochemical analyses suggest that pulmonary ECM components were preserved in comparison to the native tissue.

The acquisition and maintenance of the three-dimensional structures of various organs and tissues have been one of the primary objectives in tissue engineering [97,98]. Several studies have confirmed that biological responses occur more reliably in complex three-dimensional systems, analogous to those found in vivo, compared to two-dimensional systems [99]. The tissue structure is closely related to the spatial and functional organization of cells within the tissue microenvironment [100]. In this context, the extracellular matrix acts as a cellular signaling factor, providing molecular coordinates for cell anchorage and establishment [101,102].

The ex vivo recellularization of three-dimensional decellularized lung scaffolds has recently been investigated as an alternative approach for the treatment of pulmonary diseases [103]. This technique aims to produce functional lung tissue that, in the future, could be transplanted, representing an alternative that would allow the use of cadaveric lungs.

Suitable for biotechnological applications, an appropriate biomaterial can support and maintain viable cells, allowing for cell adhesion, migration, and proliferation [104]. The decellularized extracellular matrix (ECM) of rats’ lungs has the potential to support not only native pulmonary cells but also other cell types not physiologically found in the lung. This confirms that these scaffolds can be used as platforms for cell culture in a variety of experiments and purposes or as a source of biomaterial that can be converted into hydrogels or combined with other compounds to develop new types of biopolymers.

To form a complex system, the three-dimensional structure must be cytocompatible, providing the necessary conditions for various cell types to repopulate the scaffold and enable tissue reorganization similar to that found in vivo [98,105]. Although there are several cytocompatible biomaterials, many of them lack adhesive molecules that allow for proper cell anchorage and appropriate interaction between the cells and the biomaterial, not only at a structural level but also functionally as a signaling platform [106].

The results obtained in this study demonstrated that the scaffold fragments allowed for the adhesion of murine fibroblasts, highlighting that these biomaterials provide conditions conducive to cell survival and adhesion. Following the validation of these results, the entire lung was recellularized in a bioreactor system with mechanical ventilation for the in vitro cultivation of canine yolk sac-derived endothelial progenitor cells for the reconstruction of the pulmonary endothelium. The choice of these cells was based on their endothelial progenitor characteristics and their ability to form sprouts in functional assays, making them an important tool for producing scaffolds used in regenerative medicine, where tissue neovascularization is required.

The canine yolk sac–derived endothelial precursor cells used in this study have been previously characterized by our research group [69] and shown to exhibit angiogenic potential associated with VEGF signaling pathways. In the present study, the cells were used without additional genetic modification in order to evaluate their interaction with the decellularized lung extracellular matrix [107,108,109].

To enhance the coverage of the extracellular matrix (ECM) surface area in the vascular compartment, canine YS cells were introduced into both the arterial and venous networks of the vasculature, in line with previous studies [18,69,110]. This work demonstrates that endothelial progenitor cells treated and/or transduced with VEGF exhibit significant potential as a tool for cellular therapy in regenerative veterinary medicine, particularly regarding vascular regeneration. This approach holds promise for improving angiogenesis during tissue repair.

Bioreactors play an essential role in the bioengineering of lungs, particularly due to the intricate anatomy and physiology of the organ. These systems are vital for both artificial and biological lung scaffold fabrication, offering precise environmental control necessary for various biological processes. They facilitate tissue decellularization, recellularization, and the cultivation of cell cultures by ensuring optimal conditions for cell proliferation, differentiation, and monitoring [45,111]. By exposing re-seeded scaffolds to both physical and biochemical stimuli, bioreactors promote cellular growth and tissue regeneration, significantly impacting processes such as cell seeding, nutrient distribution, and the application of mechanical forces [112]. These stimuli are critical in guiding the development and maturation of functional lung scaffolds.

The developed bioreactor, when combined with mechanical ventilation, successfully replicated key aspects of the in vivo environment and proved to be a promising tool for pulmonary biology studies and the development of cultured lung tissue. In this context, our results are consistent with findings by Petersen et al. [74,113], Price et al. [114], and Cortiella et al. [115]. After 7 days of cultivation in the bioreactor with mechanical ventilation, these conditions supported cell survival and enhanced cell distribution in the pulmonary endothelium. The continuous motion of pulmonary structures within a bioreactor during the recellularization process, supported by controlled ventilation, plays a crucial role in enhancing nutrient distribution and waste removal [116,117]. This movement mimics the natural dynamics of the lung, promoting an optimal environment for cell survival, proliferation, and differentiation. Ventilation within the bioreactor allows for the efficient exchange of gases and distribution of culture media, facilitating the transport of essential nutrients to the cells while simultaneously dispersing metabolic waste [58]. This dynamic interaction is key to successful lung tissue engineering, as it replicates the mechanical stimuli required for the cells to adhere and grow within the scaffold, simulating the natural pulmonary microenvironment [111,118]. Several other studies have investigated the application of bioreactors in the recellularization of lung scaffolds. These studies highlight the importance of bioreactors in providing a controlled environment that mimics the physiological conditions necessary for successful cell growth and differentiation [117,119,120,121].

To investigate the potential of acellular lung scaffolds for recellularization, various bioreactors were developed and tested with different cell types. Cortiella et al. [115] employed a rotary bioreactor operating at 2 rpm, using murine embryonic stem cells (mouse ESCs) delivered via the trachea. They observed the differentiation of mouse ESCs into epithelial and endothelial lineages. Another study utilized a ventilation-based bioreactor to assess the potential of fetal mouse lung cells (FLCs) for pulmonary tissue regeneration. After an initial cell seeding via the trachea, the mouse lungs were cultured at 180 breaths/min [114], simulating the stretching induced by normal rodent respiration. This ventilation-based bioreactor supported the growth of type II alveolar fetal cells. In other attempts, a research group used a bioreactor that mimicked the fetal lung environment, including vascular perfusion and liquid ventilation, to enhance the survival and differentiation of the pulmonary epithelium. Consequently, the overall pattern of cell distribution and differentiation became more similar to native lung tissue, and gas exchange was demonstrated following the implantation of the regenerated lungs [74,113,116].

In another study, after replanting the airway compartment and the vascular network of the lung with rat fetal lung cells (FLCs) and human umbilical vein endothelial cells (HUVECs), respectively, the recellularized lung was cultured in a bioreactor at a predefined pressure range of 10–15 mmHg. This approach resulted in the reconstitution of the alveolar-capillary membrane and enabled gas exchange both in vitro and in vivo [75].

Our results demonstrated that the scaffolds, when subjected to recellularization in a bioreactor with mechanical ventilation, allowed for the adhesion of canine YSCs. This highlights that these biomaterials provide conditions conducive to cell survival, adhesion, and potentially the differentiation of stem cells into endothelial cells in the presence of VEGF-secreting cells. The interaction between the rat pulmonary extracellular matrix (ECM) and canine YS cells also indicated that the biomaterial could support non-native pulmonary cells, revealing its potential for use in various tissue engineering applications. Collectively, these findings provide substantial evidence that such scaffolds can be applied to reconstruct the pulmonary microenvironment and serve as a cell culture platform for developing in vitro models of lung tissue, drug screening assays, pulmonary cell differentiation, and even applications in other tissues, thereby opening a new realm of possibilities for ECM-derived pulmonary biomaterials. Taken together, the combination of a perfusion-based decellularization protocol that preserves major ECM components with dynamic mechanical ventilation in a whole-lung bioreactor, using yolk sac-derived endothelial precursor cells, constitutes a distinctive experimental platform. This system not only supports robust recellularization but also provides a controlled setting to investigate how lung-specific ECM and mechanical cues shape vascular regeneration and subsequent immune and barrier responses in future in vivo studies.

In addition to structural and cytocompatibility aspects, the immunological behavior of decellularized lung scaffolds represents a critical dimension for their translational use in regenerative medicine. Decellularized ECM composition and architecture modulate cellular immunity (e.g., macrophage and mast cell recruitment and polarization), humoral immunity (complement activation and antibody binding), and barrier immunity (integrity and function of the blood–air barrier and vascular endothelium). Recent reviews have emphasized that these three arms of the immune system are highly interconnected, and that subtle differences in scaffold processing, ECM preservation, and residual damage-associated molecular patterns can shift the balance between pro-regenerative and pro-fibrotic responses. In this context, our protocol, which combines efficient removal of cellular material with preservation of key ECM proteins (collagens I/III, elastin, fibronectin and laminin) and three-dimensional architecture, provides a relevant platform to investigate how lung-derived matrices influence innate and adaptive immune pathways after implantation. The dynamic recellularization of whole lung scaffolds with endothelial precursor cells under mechanical ventilation further offers an opportunity to study barrier immunity, particularly the reestablishment of an endothelial lining capable of modulating leukocyte trafficking and limiting pathological inflammation. This view is in line with recent work highlighting the need to jointly assess cellular, humoral and barrier immunity when evaluating tissue-engineered constructs and decellularized ECM scaffolds [122,123].

Despite the promising results obtained in this study, some limitations should be considered. Although successful cell adhesion and integration within the decellularized lung scaffolds were confirmed through histological analysis, DAPI staining, scanning electron microscopy, and genomic DNA quantification, endothelial lineage confirmation using specific markers such as CD31, VE-cadherin, or von Willebrand factor was not performed and should be included in future studies. In addition, while extracellular matrix preservation was demonstrated through histological and immunohistochemical analyses, quantitative biochemical assays for ECM components, such as collagen and glycosaminoglycans, were not conducted. Residual detergent levels following the decellularization process were also not directly measured, although cytotoxicity assays indicated preserved scaffold biocompatibility. Furthermore, quantitative image-based analysis of cell distribution throughout the scaffold was not performed. Future investigations addressing these aspects, as well as functional assessments of vascularization and gas exchange capacity, will further advance the development of decellularized lung scaffolds as platforms for pulmonary tissue engineering.

Although cytocompatibility and cell distribution were comprehensively evaluated in vitro, we did not assess immune responses to the decellularized or recellularized scaffolds in vivo. Consequently, key aspects of cellular immunity (e.g., macrophage and T cell infiltration and phenotype), humoral immunity (complement and antibody-mediated responses), and barrier immunity (endothelial and blood–air barrier integrity) remain to be characterized. This is a major limitation of the present work and will be specifically addressed in future studies, using implantation models designed to dissect the interplay between scaffold properties, recellularization strategies and host immune responses.

Future investigations should expand upon the present findings by incorporating endothelial-specific functional assays and in vivo implantation models to further evaluate vascular integration, immune responses, and the physiological performance of the recellularized scaffolds. In particular, assessing endothelial barrier formation, nitric oxide production, angiogenic responsiveness, and long-term scaffold integration will be essential to better characterize endothelial functionality. Additionally, in vivo studies will be necessary to determine the capacity of these bioengineered lung scaffolds to support vascular stability and gas exchange, thereby advancing their potential application in pulmonary tissue engineering and regenerative medicine.

## 5. Conclusions

In this study, we developed an effective and reproducible methodology for the decellularization of whole rat lungs using a perfusion-based approach. The resulting decellularized rat lung extracellular matrix demonstrated structural preservation and cytocompatibility, supporting the adhesion, survival, and distribution of canine yolk sac–derived endothelial precursor cells. When combined with dynamic mechanical stimulation in a bioreactor system, the scaffold provided a favorable microenvironment for cellular integration within the lung architecture. These findings highlight the potential of decellularized rat lung ECM as an in vitro platform for investigating endothelial precursor cell behavior and recellularization strategies in pulmonary tissue engineering.

Beyond providing a structurally preserved and cytocompatible scaffold for endothelial precursor cells, our model establishes a foundation to systematically investigate how lung-derived ECM and dynamic mechanical stimulation influence immune and barrier responses after implantation. Future in vivo studies building on this platform will be essential to determine whether the observed in vitro performance translates into favorable cellular, humoral and barrier immunity, ultimately supporting long-term graft integration and function.

## Figures and Tables

**Table 1:** Summary of the whole-lung perfusion decellularization protocol, including solution composition, concentration, perfusion route, duration, and perfusion parameters.

Step	Solution	Concentration	Perfusion Route	Duration	Flow Rate (Trachea and Artery)	Pressure
Initial washing	dH_2_O	—	Trachea + artery	1 h	2 mL/min via tracheal and 4 mL/min via arterial	10 cmH_2_O via tracheal and 20 cmH_2_O via arterial
PBS wash	PBS	1×	Trachea + artery	1 h	2 mL/min via tracheal and 4 mL/min via arterial	10 cmH_2_O via tracheal and 20 cmH_2_O via arterial
Decellularization	SDS + Triton X-100	SDS 0.5% + Triton X-100 0.5%	Trachea + artery	5 h	2 mL/min via tracheal and 4 mL/min via arterial	10 cmH_2_O via tracheal and 20 cmH_2_O via arterial
Final washing	dH_2_O	—	Trachea + artery	6 h	2 mL/min via tracheal and 4 mL/min via arterial	10 cmH_2_O via tracheal and 20 cmH_2_O via arterial
Final wash	PBS	1×	Trachea + artery	6 h	2 mL/min via tracheal and 4 mL/min via arterial	10 cmH_2_O via tracheal and 20 cmH_2_O via arterial

## Data Availability

The original contributions presented in this study are included in the article. Further inquiries can be directed to the corresponding author.
